# Cognitive-Behavioral Therapists’ Experience on Relevance of Sleep and Sleep Disorders in Training and Clinical Practice: A Survey Study from Italy

**DOI:** 10.3390/brainsci15010048

**Published:** 2025-01-07

**Authors:** Chiara Baglioni, Andrea Galbiati, Debora Meneo, Greta Cavadini, Francesca Gelfo, Francesco Mancini, Carlo Buonanno

**Affiliations:** 1 Department of Human Sciences, Guglielmo Marconi University, 00193 Rome, Italy; c.baglioni@unimarconi.it (C.B.); d.meneo@unimarconi.it (D.M.); f.gelfo@unimarconi.it (F.G.); f.mancini@unimarconi.it (F.M.); 2 Department of Psychiatry and Psychotherapy, Medical Center, Faculty of Medicine, University of Freiburg, 79104 Freiburg, Germany; 3 Faculty of Psychology, Vita-Salute San Raffaele University, 20132 Milan, Italy; g.cavadini3@studenti.unisr.it; 4 Department of Clinical Neurosciences, Neurology-Sleep Disorders Center, IRCCS San Raffaele Scientific Institute, 20132 Milan, Italy; 5 IRCCS Fondazione Santa Lucia, 00179 Rome, Italy; 6 School of Cognitive Psychotherapy, Association of Cognitive Psychotherapy, 00185 Rome, Italy; buonanno@apc.it

**Keywords:** insomnia disorder, sleep-wake disorders, cognitive behavioral therapy, CBT, survey

## Abstract

Background/Objectives: Based on previous data reporting the status of health professionals’ training about sleep clinical psychophysiology, insomnia, and its treatment in the US and Canada, this paper aims at providing a snapshot of the Italian situation, considering health professionals qualified to offer cognitive behavioral therapy (CBT). Adding information on different countries is important, as national health systems differ significantly, and distinct evidence-based pathways for change may be proposed. Methods: Two hundred and thirteen CBT professionals (180 females; 33 males) answered a 5 min survey about their training and experience in recognizing and treating behavioral sleep disorders in their practice. The questionnaire was diffused through the mailing list of the Italian Behavioral and Cognitive Therapy Society (Società Italiana di Terapia Comportamentale e Cognitiva, SITCC) throughout December 2023 and January 2024. Results: A total of 213 participants completed the survey. Only a minor proportion of respondents (37.1%) reported having received training for diagnosis and treatment of insomnia or other behavioral sleep disorders. Familiarity with psychological therapeutics for sleep was mainly associated with knowledge of sleep hygiene rules, relaxation, and mindfulness techniques, but not with core CBT strategies for insomnia (i.e., sleep restriction and stimulus control) and sleep regulation. The less familiar therapeutics were those for pediatric insomnia. Conclusions: The results of this study highlight scarce knowledge and consideration of sleep problems in CBT practice in Italy. As insomnia is prevalent, an independent mental disorder, and a predictor for mental and somatic comorbid conditions, these findings underscore an urgency to enlarge and strengthen CBT professionals’ training on sleep psychophysiology, sleep clinical psychology, insomnia, behavioral sleep problems, and their treatment.

## 1. Introduction

Insomnia has been observed to be the second most common mental disorder in western countries [1], and it is associated with high direct and indirect social costs [2]. Moreover, numerous meta-analyses have shown that individuals with insomnia are more vulnerable to developing various mental and somatic disorders [3,4]. Evidence-based clinical guidelines for the evaluation and treatment of insomnia for Europe, the US, the UK, Australia, and Asian countries [2,5,6,7,8,9] agree that cognitive behavioral therapy (CBT) is the first-line intervention for the disorder. Nevertheless, research and clinical data evidence that:

- insomnia remains underdiagnosed and undertreated in most countries;
- when diagnosed, insomnia is mainly treated with pharmacological therapies in most countries; and
- psychological courses and CBT specialization courses seldom offer training on sleep and insomnia.

As a result, most patients in most countries do not access the best evidence-based therapy they need. Earlier studies conducted in the US and Canada revealed limited knowledge and insufficient professional training among psychologists and CBT practitioners regarding sleep and insomnia [10,11]. Because of several important differences between local national health systems (for a review, see [12]), it is important to share knowledge on the specific situation in each single nation to promote general and country-specific actions aimed to disseminate the best evidence-based treatments around the world as much as possible. Cultural differences may significantly impact outcomes, making it essential to consider tailored, evidence-based approaches for implementing changes in different countries. In the present paper, which reports the findings obtained in a survey study, we want to provide a snapshot of Italian CBT professionals’ training and practice related to sleep behavioral disorders.

### 1.1. Insomnia: A Highly Prevalent Mental Disorder

Insomnia disorder is prevalent and costly, and it represents a risk factor for several mental and somatic disorders [2,3,4,13,14]. The prevalence of insomnia in the general population is estimated to be around 10% (e.g., [2]), with evidence suggesting an increase following the Covid-19 pandemic [15,16]. Despite having a significant impact on health and quality of life, poor sleep and insomnia still receive scarce attention, and results from numerous studies indicate that they often go underdiagnosed and undertreated [12,17,18]. Patients with mental disorders commonly report physiological and subjective complaints of sleep continuity [19] and a higher prevalence of insomnia [20,21,22,23]. Comorbid insomnia is often associated with poor resolution of the concomitant problem [24,25,26]. Although insomnia is an independent diagnosis, its frequent comorbidity with other conditions complicates the diagnostic process, often requiring the involvement of multiple professionals. Since the publication of the fifth edition of the Diagnostic and Statistical Manual of Mental Disorders (DSM-5) [27], insomnia has been recognized as an independent mental disorder, which follows its own course and responds to specific treatment whether it presents alone or in comorbidity. According to scientific evidence, the first line treatment of the disorder is cognitive behavioral therapy for insomnia (CBT-I) [2]. Furthermore, data indicate that patients with complex, chronic psychiatric conditions can obtain sleep improvements with CBT-I beyond those obtained with pharmacotherapy alone [28]. Nevertheless, despite these strong recommendations, data from various sources indicate that at present CBT-I is offered to a very small proportion of patients with insomnia [17,29]. Conversely, pharmacotherapy is still by far the most prevalent intervention for insomnia in routine healthcare worldwide (e.g., [2,12,30]. The World Health Organization points out that it is a human right to receive the most evidence-based treatment; thus, authors point out that it is a worldwide priority to diffuse CBT-I [30].

### 1.2. Cognitive Behavior Therapy for Insomnia Disorder (CBT-I): What Is It?

Following the definition by Espie [31], CBT-I is a system of therapeutics, rather than a specific treatment. CBT-I is thus an umbrella term for all those evidence-based therapeutics, including behavioral, cognitive, emotional, motivational, and psychoeducational interventions, aimed at improving sleep quality and daytime associated symptoms. Those therapeutics are briefly described in Table 1.

The minimal characteristics of the standard CBT-I should include at least one effective behavioral intervention and one effective cognitive intervention [9,31]. Efficacy data [32] is based on more than 100 randomized controlled trials and 25 meta-analyses [2]. Studies indicate that CBT-I is highly effective in addressing nighttime symptoms of insomnia, with moderate to large effect sizes, and somewhat less effective for daytime symptoms. CBT-I is effective in treating insomnia also in patients presenting comorbid mental disorders [2]. Recently, digital treatment has also been recommended for insomnia disorder for its efficacy and ability to reach out a great number of individuals [2,30], though face-to-face intervention shows larger effects compared to digital CBT-I [33]. Numerous research studies have evidenced that CBT-I is superior to pharmacotherapy, especially for long-term outcomes, and should be the first-line intervention for insomnia disorder [2].

As Espie [31] points out, therapeutics offered to a specific patient should be based on a formulation-driven approach, where case conceptualization leads to the personalized selection of CBT components and the ways in which they are introduced and delivered. In other words, health practitioners offering CBT-I should be well trained in (e.g., [12,34,35]):

(1) CBT-I principles and practice;
(2) CBT-I case conceptualization and formulation driven treatment;
(3) Sleep psychophysiology and insomnia disorder; and
(4) Specific CBT therapeutics for insomnia disorder.

### 1.3. The Size of the Problem

Despite growing media attention, several barriers have been identified as limiting the widespread offer of CBT-I to patients [17]. Of these, two main aspects should be considered. First, evidence has pointed out that both patients and clinicians have poor knowledge of those factors that cause and maintain insomnia (e.g., [17]). From the patient’s perspective, insomnia is often seen as a stable trait, something you are born with. Therefore, individuals with insomnia tend not to seek help for their sleep problem. From the clinician’s perspective, most clinical psychologists, psychotherapists, and psychiatrists, as well as medical doctors from other specializations, still know relatively little about the diagnosis and treatment of sleep disorders, including insomnia (e.g., [36,37]). This can lead to misconceptions from both patients and clinicians about the treatment course for CBT-I (e.g., typically around 6 weeks active treatment with 1 h weekly sessions, in contrast to months of psychotherapy) [10,12].

CBT-I evidence-based non-pharmacological interventions are not routinely offered in primary care; even when a patient does seek help, insomnia is often poorly evaluated and may not even be diagnosed or treated. Furthermore, particularly for those in countries where treatment is not provided as primary care intervention, psychological treatment and psychotherapy may be costly for a patient, perhaps prohibitively so.

Nevertheless, as a treatment approach, CBT-I has several advantages:

1. Compared with psychological treatment or psychotherapy for mood and anxiety disorders, which may be generally longer-term, CBT-I is a relatively short intervention with an average of 6 to 10 sessions (e.g., [6]).
2. While short-term costs may be higher than medication, both in terms of money and clinician time, CBT-I has been reported to be more cost-effective overall compared to pharmacotherapy [38] and has a much safer and more favorable side-effects profile. It promotes the development of personal resilience and self-regulatory skills and whereas the effects of medication do not usually last beyond the treatment period, effects of CBT-I have been shown to last up to 10 years after treatment (e.g., [39,40,41]).
3. As not all patients will require a comprehensive and individually tailored therapeutic intervention delivered by an expert clinician, there is the opportunity for care to be provided via structured clinical management (SCM) (see [6,12,42]). Indeed, short-term, manualized treatment protocols or digital interventions may already be adequate for a large proportion of patients [30,43].

### 1.4. Current Status of Professionals’ Training in CBT-I

Increasing the number of CBT-I experts is of utmost importance given the lack of trained providers, which hinders the proper and timed treatment of the vast majority of patients affected by chronic insomnia [12]. Following the stepped-care model for insomnia [18,42], CBT-I could be administered in steps to reach many patients. Self-help treatment without consistent support from a therapist might constitute the first step. Vice versa, face to face CBT-I should be administered to those patients with mental or sleep disorder comorbidity by a specialist in clinical sleep psychophysiology and behavioral sleep health practice, representing the highest step where manualized treatments are not sufficient. Currently, evidence suggests that training at the foundation level (first step) is a priority since there is a clear lack of adequate knowledge regarding assessment, diagnosis, and treatment of the disorder [44]. A recent work by Jernelöv and Blom [45] aimed to describe and evaluate the training of CBT-I professionals at the international level. They systematically reviewed 14 papers describing and evaluating existing training in the USA, Australia, UK and Portugal. The authors confirmed the systematic lack of CBT-I training for professionals at all educational and professional levels. In general, when training programs are provided, trainees appreciate CBT-I training and the development of relevant skills is clearly detectable. Importantly, there are some limitations in the generalizability of these data. Indeed, regulations and requirements regarding who may deliver CBT-I are different between countries. For example, in some countries, such as Great Britain, nurses are allowed to administer CBT-I, and this choice is supported by recent results [46]. Conversely, in others, such as Italy, only licensed psychologists or psychotherapists are allowed to administer this treatment. Taken together, all these considerations encourage the development of a high-quality training and evaluation system taking into account each specific context to provide data on which type of training is effective.

### 1.5. Previous Studies in the United States and in Canada

In 2009, a groundbreaking study [10] interviewed 212 directors of undergraduate and graduate Psychology programs in the United States and Canada, focusing on the inclusion of sleep-related teachings. The results revealed that merely 6% of the evaluated programs had specific courses dedicated to sleep. A subsequent study conducted in 2020 [11] delved into the practices of 200 clinical psychologists practicing in both the United States and Canada. These professionals were reached through mailing lists of national psychology associations. The questionnaire aimed to gather information, regarding:

- The professional’s title, type of psychology degree, years since licensure, years of clinical practice, and region of practice;
- The types of patients they treat, considering age and clinical characteristics;
- The number of hours of sleep-related lectures during their undergraduate or postgraduate master’s program;
- Their self-assessment of preparedness in addressing sleep-related issues, rated on a 5-point Likert scale ranging from “not prepared” to “very prepared”;
- The therapeutic approach employed for sleep problems; and
- Their expectations and aspirations concerning this vital topic for the future.

One striking finding was that the interviewed specialists reported a median of 10 h of total teaching focused on specific sleep-related subjects during their pre- and post-graduate professional training. Surprisingly, a staggering 95% of them disclosed that they did not receive any dedicated clinical psychology module specifically addressing sleep during their training. Nevertheless, 63% of the participants expressed feeling at least “moderately prepared” in evaluating sleep issues in their patients, and 59.5% felt “moderately prepared” in treating such problems. Despite these moderately positive self-assessments, the majority of respondents confessed to offering therapeutic interventions that did not align with international guidelines on effective treatments for sleep behavioral disorders.

To date, however, no studies have evaluated the training and clinical experience of the main reference professionals: psychotherapists with a cognitive-behavioral orientation.

### 1.6. Objectives

Building upon the reviewed literature, the objective of this study is to assess, in an explorative manner, the current state of training and clinical practice for cognitive-behavioral psychotherapists in Italy as it relates to the treatment of sleep disorders.

By examining the existing educational landscape and its possible impact on clinical practice, this study seeks to identify any gaps or limitations in the Italian healthcare system’s ability to provide effective care for individuals with behavioral sleep disorders. This was accomplished by assessing the educational and clinical background of Italian cognitive-behavioral oriented psychotherapists in relation to sleep-related issues. The methodology involves surveying registered psychotherapists through the Italian Society of Cognitive and Behavioral Therapy (SITCC) to gather demographic and clinical practice data, as well as information on their training on sleep-related issues.

## 2. Materials and Methods

### 2.1. Sample Characteristics

The study’s sample consists of the population of Italian psychotherapists and psychotherapists in training who are registered members of the Italian Society of Cognitive Behavioral Therapy (SITCC: https://www.sitcc.it/).

### 2.2. Recruitment

Participants were recruited through the mailing list of SITCC. The email, sent on 7 December 2023, contained a brief description of the study and a direct link to access the online questionnaire. Upon clicking the link, those interested could view an informative page and, as a necessary condition to continue their compilation, provide their consent for participation. Data were collected in a completely anonymous form. A follow-up email was sent on 8 January 2024 to encourage participation. After 2 months of recruitment, 213 responses were collected, and these served as the study’s final sample. This sample size was comparable to the one of a previous study on clinical psychologists [11].

### 2.3. Assessment Tools

The questionnaire, which took approximately 5 min to complete, collected demographic data such as gender and age, followed by information on academic and professional background, including the years since graduation and specialization as a psychotherapist. Years of experience as a psychotherapist was assessed with a specific question; the response option “I’m still in training” was used to categorize participants with no clinical experience. The questionnaire then proceeded to explore current clinical practice, the number of patients treated per year, and the type of patients psychotherapists typically worked with. For questions pertaining to actual clinical practice, participants could also choose an option indicating “No current clinical practice”. The next section focused on the participants’ self-efficacy, asking them to assess their readiness and confidence to evaluate and treat sleep-wake disorders. Lastly, the questionnaire concluded with questions about training experience regarding psychological interventions for sleep disorders and the importance of addressing such issues in a psychotherapeutic context. 

### 2.4. Informed Consent

To participate in the study, respondents first reviewed the study information and provided their explicit consent. Upon clicking the provided link, interested individuals were redirected to an informative page that delineated the study’s objectives, procedures, and expected outcomes. Here, participants had the opportunity to carefully review this information and make an informed decision about their participation. Absolute anonymity was ensured for all participants. The data collected were processed in strict accordance with the European General Data Protection Regulation (GDPR) of 25 May 2018 and also complied with D.L. 8 October 2021 n. 139, converted, with amendments, by L. 3 December 2021 n. 205.

### 2.5. Statistical Analysis

Descriptive statistics were performed, reporting frequency, mean, standard deviation, median, and interquartile range. We reported the sample characteristics and clinical practice, formal training received with respect to sleep difficulties, perceived preparation in evaluating and treating insomnia and other sleep disorders, level of familiarity with CBT-I techniques, and type of treatment approach used. We descriptively compared the self-efficacy (perceived preparation) between (a) different levels of clinical experience and (b) formal training received (yes/no), by tabulating the mean and standard deviations and median and interquartile range of the rating in perceived preparation (from 1 = not prepared to 5 = very prepared). We reported familiarity with CBT-I techniques and descriptively compared the rates of reporting to be familiar or very familiar with each technique between (a) different levels of clinical experience (years of experience) and (b) frequencies of addressing sleep difficulties in patients (never, sometimes, always).

## 3. Results

### 3.1. Sample Characteristics and Clinical Practice

The study included 213 participants, comprising 180 females and 33 males, all of whom were members of the SITCC. Younger participants were more prevalent: 26.8% fell within the age range of 30–34 years, while 18.3% were distributed across the age groups of 35–39 and 40–44. Concerning clinical background, a significant proportion (26.8%) had graduated 6–10 years prior, whereas 28.6% were undergoing specialization training during the survey. Experience-wise, the majority (27.7%) held 1–5 years of practice, catering to 10–30 patients annually (34.3%). Interestingly, a sizeable portion (24.9%) focused on treating children and adolescents, while anxiety disorders (69%), mood disorders (50.2%), and personality disorders (39.4%) emerged as the most prevalent conditions encountered. Sleep-wake disorders were reported by 41 participants as a specific mental health problem presented by patients (19.2%).

In relation to the specific scope of the study, respondents noted that approximately 20–30% of their patients experienced sleep difficulties (24.9%), with challenges in sleep maintenance (79.8%) and sleep initiation (65.3%), core symptoms of insomnia disorder, ranking among the foremost issues observed (see Table 2). Only eight participants indicated a 0% prevalence of sleep difficulties (3.8%). Data are reported in Table 1.

### 3.2. Perceived Preparation

Among the 213 participants, 79 (37.1%) reported that they had received specific training on psychological interventions for sleep difficulties. Among them, 13 participated in an accredited post-graduation course from the European Sleep Research Society (ESRS) CBT-I Academy (https://esrs.eu/research-networks/european-insomnia-network/ accessed on 12 December 2024) and/or the Italian Association of Sleep Medicine (https://sonnomed.it/).

When asked about their perceived preparation to evaluate and treat insomnia disorder in their patients, one-third reported that they were “Prepared” or “Very prepared” to assess the disorder (30.1%), and less than one-third reported their preparation to treat it (21.2%). The perceived preparation to evaluate and treat other sleep-wake disorders was generally lower than for insomnia disorder (see Table 3 for details).

We computed the mean and median of the rating of perceived preparation (from 1 = not prepared to 5 = very prepared) to descriptively compare the self-efficacy in the evaluation and treatment of sleep-wake disorders between more and less experienced psychotherapists and between those who had received formal training in sleep difficulties and those who had received no training. Given the small number of participants in some experience groups, they were aggregated into three groups: no experience, 5 years or less, and more than 5 years. The self-efficacy in the evaluation and treatment of insomnia disorder and other sleep-wake disorders was higher in more experienced psychotherapists and those reporting specific training compared to no training (see Table 4). The differences were narrower for other sleep-wake disorders as compared to insomnia disorder.

### 3.3. Attitudes Toward the Treatment of Sleep Difficulties

Regarding treatment approaches, the vast majority regarded it as important to address sleep difficulties during psychotherapy (82.6%). Less than one-third always addressed sleep difficulties in their patients, while the majority reported sometimes addressing them. A total of 10.8% of therapists reported never addressing sleep difficulties in their patients (see Table 5). The most used treatment approach was sharing sleep hygiene rules (72.8% of responders), followed by sharing behavioral advice, such as regular physical activity (63.8%), and prescribing relaxation techniques (62%) (see Table 5). CBT-I was applied by 28.2% of psychotherapists. Among those reporting to share sleep hygiene rules, only 32 (20.6%) also applied CBT-I.

**Figure 1:**
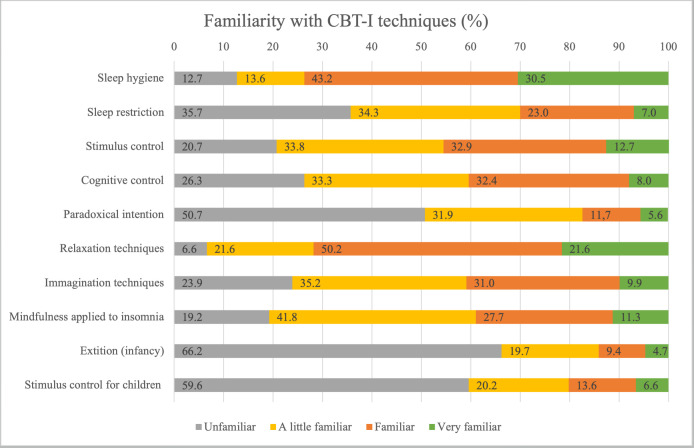
Familiarity with CBT-I therapeutics reported by respondents.

Participants were asked to rate their familiarity with CBT-I therapeutics, which included the core components of CBT-I, adjunctive therapeutics (mindfulness), and techniques used to treat pediatric insomnia in young children (bedtime routines, extinction). Overall, familiarity was higher for sleep hygiene and relaxation techniques, with 73.7% and 71.7% of psychotherapists reporting to be familiar or very familiar with sleep hygiene and relaxation, respectively. The less familiar therapeutics were those for pediatric insomnia (see Figure 1).

Subgroups of participants based on clinical experience (no experience, 0 to 5 years, more than 5 years) and on frequency of addressing sleep difficulties in their patients (never, sometimes, always) were descriptively compared regarding the rate of reporting to be familiar or very familiar with CBT-I techniques (see Table 6). The rate of familiarity with CBT-I techniques increased with experience, except for techniques adopted in childhood. A low proportion of those who never addressed sleep difficulties were familiar or very familiar with core CBT-I techniques. For instance, among those who never addressed sleep difficulties, 17.4% and 21.7% reported being familiar or very familiar with sleep restriction and stimulus control, respectively, compared to a prevalence of 50.9% and 70.9% among those reporting that they always addressed sleep difficulties.

## 4. Discussion

This study represents the first attempt to examine the current state of CBT professionals’ perceptions of their training and clinical practices in Italy regarding sleep-related behavioral problems. Consistent with expectations, the majority of respondents reported limited training and inadequate knowledge of sleep-related factors relevant to psychological treatment.

Summarizing the main findings:

(1) Professionals underscored the widespread diffusion of behavioral sleep difficulties in patients with mental disorders, likely demonstrating that these symptoms are often overseen in clinical practice.
(2) CBT practitioners frequently lack training in identifying and managing sleep and insomnia-related difficulties.
(3) Most familiar CBT interventions referred to sleep hygiene and relaxation techniques, which however are considered not standalone therapy for insomnia (e.g., [31]).
(4) The most prevalent treatment approach for behavioral sleep difficulties in psychological treatments was sharing sleep hygiene rules, which is actually against evidence, as nowadays, sleep hygiene is not considered an effective intervention for insomnia disorder [9,32].

Sleep difficulties were reported as prevalent in the psychotherapeutic setting. However, the estimation made by the professionals in this study was lower than the estimated prevalence of insomnia in mental disorders. Indeed, the majority of respondents reported that the prevalence of sleep disorders was below 30% in their clinical practice. This percentage was well below what is reported by studies evaluating comorbidity. This discrepancy may be partly explained by confounding factors such as the perception of CBT-I as a less viable option for managing insomnia, both among healthcare professionals and patients. Limited professional training and patient awareness could lead to underdiagnosis and undertreatment of sleep disorders, contributing to the observed gap between clinical practice and epidemiological data. As an example, Geoffroy et al. [21] reported that patients with a diagnosis of major depressive disorder had insomnia or hypersomnia in 85.2% and 47.5% of cases. Moreover, comorbidity between insomnia and social anxiety disorder has been reported in between 26% and 70% of patients, and between 30.8% and 70% in obsessive compulsive disorder [47]. The discrepancy between what is reported in the literature and what is perceived by therapists probably reflects the tendency of clinicians to primarily treat anxiety and mood disorders, which are highly comorbid with insomnia disorder. Furthermore, current standards in psychotherapy do not include questions on sleep pattern nor specific screening questionnaires about sleep health. Most of the sample was composed of young participants with 1 to 5 years of professional experience. Few reported formal training in the assessment and treatment of sleep difficulties. This can further reduce self-efficacy in assessing and treating sleep difficulties, which in our sample was higher among trained and more experienced psychotherapists. Moreover, most CBT psychotherapists were familiar with non-specific CBT-I therapeutics, such as sleep hygiene, relaxation, and mindfulness, while the overall familiarity with core CBT-I therapeutics, such as stimulus control and sleep restriction, was poor. These data further support the need to increase sleep training as part of the clinical training of CBT psychotherapists. In the clinical practice of the studied therapists, the most used interventions were sleep hygiene and relaxation, which are not recommended as stand-alone treatments for insomnia disorder [9]. Few of those using sleep hygiene education did so in association with CBT-I.

While CBT professionals should be the first indicated professionals to offer CBT-I, they are often poorly educated in their professional careers about sleep psychophysiology and sleep clinical psychology. This result in difficulties with insomnia going underdiagnosed and undertreated in clinical practice. In addition, when anything is offered, professionals are most likely to prescribe therapies which are against evidence, such as sleep hygiene rules. According to the guidelines provided by a task force of the American Academy of Sleep Medicine for behavioral and psychological treatments for chronic insomnia disorder [9], sleep hygiene received a conditional recommendation against its use as a single component. In spite of this, the task force remarked that sleep hygiene may be included when providing multicomponent CBT-I. On the other hand, stimulus control and sleep restriction are indicated as standalone treatments, along with relaxation techniques, again with a conditional recommendation. The only strong recommendation was received for the administration of CBT-I as a multicomponent intervention. Considering these recommendations, in our sample, therapists were shown to be familiar with a therapeutic approach that is not suggested as an efficacious treatment (i.e., sleep hygiene), and on the other hand, they were shown to have poor knowledge regarding the core behavioral components of the disorder that are recognized to be effective as standalone therapeutics (i.e., sleep restriction and stimulus control). Therefore, there is an urge to add high quality sleep/insomnia training in CBT careers to increase the number of professionals who can provide evidence-based best treatments for the disorder.

Another relevant aspect emerged from the survey regarding the treatment of sleep disorders in the pediatric population. Among respondents, 24.9% reported dealing with children and adolescents in their clinical practice, but only a low percentage of therapists were familiar with techniques targeting sleep problems in this population (i.e., extinction techniques for infants, and stimulus control for children). A recent meta-analysis [48], aimed at testing the efficacy of non-pharmacological treatments for sleep initiation and maintenance problems in healthy pediatric populations, reported that evidence-based psychological interventions are effective in reducing symptoms. In this field, there is also a need for trained professionals, given the high prevalence of unhealthy sleep patterns found among Italian children and adolescents [49]. Considering that Mombelli and collaborators [48] concluded that there is a lack of randomized controlled studies for pediatric insomnia, it is of utmost importance to boost the interplay between clinical professionals and research in this field.

The training of CBT-I professionals on a global scale has been the focus of a recent study [45] evaluating this aspect in the USA, Australia, the UK, and Portugal. The authors found a consistent lack of CBT-I training for professionals across all educational and professional levels. Generally, when training programs are available, trainees value the CBT-I training, and significant skill development is observed. However, the generalizability of these findings is limited due to varying regulations and requirements regarding who can deliver CBT-I across different countries. For instance, in some countries, nurses are permitted to administer CBT-I, supported by recent findings [46], while in others, only licensed psychologists or psychotherapists are allowed to provide this treatment. From this perspective, the Italian situation needs to be paid particular attention, as CBT is allowed to be practiced exclusively by clinical psychologists, psychotherapists, and psychiatrists, and thus, Italian data are not comparable with data coming from other countries. Furthermore, integrating the patient’s perspective into the delivery of CBT-I is equally important. Understanding how patients perceive and engage with sleep treatments could improve treatment adherence, tailor interventions to individual needs, and enhance the overall effectiveness of CBT-I. As such, expanding training programs to include patient-centered approaches, alongside professional competencies, would significantly improve the quality and success of CBT-I across various healthcare systems.

## 5. Conclusions

This study highlights the significant gap in CBT professionals’ training and clinical practices regarding sleep-related behavioral problems in Italy. Despite the widespread prevalence of insomnia in patients with mental disorders, these issues are often underdiagnosed and undertreated. Many practitioners rely on outdated or unsupported interventions, such as sleep hygiene education alone, instead of evidence-based CBT-I techniques. The lack of formal training in core CBT-I methods like stimulus control and sleep restriction underscores the need for better professional education. Addressing these deficiencies is critical to aligning clinical practices with established guidelines and improving treatment outcomes. Pediatric sleep disorders also remain inadequately managed due to limited familiarity with effective interventions. Boosting collaboration between clinicians and researchers could advance evidence-based approaches for both adults and children. Expanding training programs, including patient-centered methods, can further enhance adherence and efficacy. The Italian situation warrants particular attention given its unique regulatory context, making targeted efforts to standardize and improve CBT-I training essential. Ultimately, equipping CBT professionals with the skills to address sleep difficulties can significantly impact mental health care quality.

## Figures and Tables

**Table 1:** Cognitive behavioral therapy for insomnia (CBT-I) therapeutics.

Therapeutics	Categorization of the Therapeutics	Description of the Therapeutics
Sleep restriction (or bedtime restriction)	Behavioral intervention	Aims at enhancing the body’s natural sleep regulation mechanisms, promoting a balanced sleep-wake cycle and stable circadian rhythms, by reducing the chance of sleeping over consecutive nights.
Stimulus control	Behavioral intervention	Guidelines rooted in the operant conditioning paradigm which aim to assist individuals with insomnia in establishing a consistent sleep routine, reduce the association of the bed with activities that could disrupt sleep, and reinforce the bed as a cue for sleep.
Cognitive control	Cognitive intervention	The patient is advised to make a list of concerns and tasks for the following day while seated comfortably in an armchair with the aim of addressing anxieties beforehand, thus reducing emotionally charged intrusive thoughts that may disrupt the process of falling asleep.
Relaxation	Behavioral intervention	A set of techniques, comprising progressive muscle relaxation, autogenic training, imagery training, and meditation, designed to reduce heightened physical or mental arousal.
Imagery	Cognitive intervention	Mental visualization techniques to create or manipulate mental images that can affect emotions, behaviors, or perceptions.
Sleep hygiene education	Psychoeducational intervention	Guidance and recommendations for optimizing sleep habits and environment to promote better sleep quality.
Sleep Health Dimensions education	Psychoeducational intervention	Comprehensive approach to educating individuals about various aspects of sleep that contribute to overall well-being.
Mindfulness for insomnia	Cognitive and Emotional Intervention	Mindfulness techniques to manage sleep problems by focusing on the present moment without judgment.
Extinction therapies (for the developmental age)	Behavioral intervention	Behavioral techniques aimed at reducing or eliminating unwanted behaviors by removing reinforcement or rewards previously associated with those behaviors.
Bedtime routines (for the developmental age)	Behavioral intervention	Structured activities and rituals designed to help children transition from wakefulness to sleep.

**Table 2:** Demographic characteristics and clinical activity of the sample.

	N	%		N	%
**Gender**			**Specific populations**		
Male	33	84.5	Children and adolescents	53	24.9
Female	180	15.5	Pregnant and peripartum women	9	4.2
**Age**			Patients with medical conditions	36	16.9
<30	22	10.3	Patients with disabilities	15	7.0
30–34	57	26.8	Patients over 60 years	16	7.5
35–39	39	18.3	No specific population	133	62.4
40–44	39	18.3	No clinical activity	5	2.3
45–49	21	9.9	**Specific mental disorder**		
50–54	13	6.1	Mood disorders	107	50.2
55–59	9	4.2	Anxiety disorders	147	69.0
60–64	5	2.3	Obsessive-compulsive disorder	79	37.1
65–69	6	2.8	Trauma and stress	65	30.5
70–74	2	0.9	Eating disorders	49	23.0
**Years from graduation**			Sleep-wake disorders	41	19.2
<5	34	16.0	Sexual disfunction	14	6.6
6–10	57	26.8	Conduct disorder	35	16.4
11–15	44	20.7	Substance-related disorders	30	14.1
16–20	36	16.9	Neurocognitive disorders	26	12.2
21–25	20	9.4	Personality disorders	84	39.4
26–30	9	4.2	Others	8	3.8
31–35	5	2.3	No specific disorder	42	19.7
36–40	5	2.3	No clinical activity	4	1.9
>40	3	1.4	**Prevalence sleep difficulties**		
**Years from specialization**			0%	8	3.8
In training	61	28.6	Less than 10%	42	19.7
<1	12	5.6	10–20%	46	21.6
1–5	46	21.6	20–30%	53	24.9
6–10	34	16.0	40–60%	37	17.4
11–15	30	14.1	60–80%	16	7.5
>15	30	14.1	More than 80%	4	1.9
**Years of experience**			100%	2	0.9
In training	39	18.3	Don’t know	1	0.5
<1	15	7.0	No clinical activity	4	1.9
1–5	59	27.7	**Type of sleep difficulty ^†^**		
6–10	35	16.4	No difficulties	6	2.8
11–15	35	16.4	Difficulty in sleep initiation	139	65.3
16–20	14	6.6	Difficulty in sleep maintenance	170	79.8
21–25	7	3.3	Difficulty in early awakening	75	35.2
26–30	5	2.3	Excessive daytime sleepiness	49	23.0
31–35	3	1.4	Frequent nightmares	35	16.4
36–40	1	0.5	Circadian rhythm disruption	30	14.1
**Annual number of patients**			Night binge eating	19	8.9
No clinical activity	6	2.8	Parasomnias	12	5.6
<10	33	15.5	No clinical activity	5	2.3
10–30	73	34.3			
31–50	50	23.5			
>50	51	23.9			

^†^ Multiple choices.

**Table 3:** Perceived preparation in the assessment and treatment of insomnia and other sleep disorders.

	Not Prepared		A Little Prepared		Moderately Prepared		Prepared		Very Prepared	
	n	%	n	%	n	%	n	%	n	%
**Insomnia**										
Assessment	14	6.6	55	25.8	80	37.6	50	23.5	14	6.6
Treatment	37	17.4	60	28.2	71	33.3	34	16.0	11	5.2
**Other sleep-wake disorders**										
Assessment ^†^	23	10.9	78	37.0	69	32.7	31	14.7	10	4.7
Treatment	35	16.4	89	41.8	58	27.2	29	13.6	2	0.9

^†^ Missing data = 2.

**Table 4:** Descriptive comparison of self-efficacy in the assessment and treatment of insomnia and other sleep disorders.

		Insomnia Disorder				Other Sleep Disorders			
		Assessment		Treatment		Assessment ^†^		Treatment	
	N	M (SD)	Median (IQR)	M (SD)	Median (IQR)	M (SD)	Median (IQR)	M (SD)	Median (IQR)
**Total sample**	213	2.98 (1.01)	3.00 (2.00–4.00)	2.63 (1.1)	3.00 (2.00–3.00)	2.65 (1.01)	3.00 (2.00–3.00)	2.41 (0.95)	3.00 (2.00–3.00)
**Years of experience**									
No experience	61	2.33 (0.95)	2.00 (2.00–3.00)	1.97 (0.93)	2.00 (1.00–2.50)	2.18 (0.85)	2.00 (2.00–3.00)	1.92 (0.81)	2.00 (1.00–2.00)
0–5 years	58	2.89 (1.03)	3.00 (2.00–3.00)	2.55 (1.12)	2.50 (2.00–3.00)	2.68 (1.08)	3.00 (2.00–3.00)	2.36 (0.90)	2.00 (2.00–3.00)
More than 5 years	94	3.29 (0.89)	3.00 (3.00–4.00)	2.95 (1.03)	3.00 (2.00–4.00)	2.82 (0.97)	3.00 (2.00–3.00)	2.63 (0.97)	2.50 (2.00–3.00)
**Specific training**									
No training	134	2.57 (0.83)	3.00 (2.00–3.00)	2.15 (0.85)	2.00 (2.00–3.00)	2.29 (0.80)	2.00 (2.00–3.00)	2.05 (0.77)	2.00 (2.00–2.00)
Training	79	3.67 (0.92)	4.00 (3.00–4.00)	3.46 (1.00)	3.00 (3.00–4.00)	3.27 (1.05)	3.00 (2.00–4.00)	3.01 (0.93)	3.00 (2.00–4.00)

**Note**: Perceived preparation was assessed on a scale from 1 = not prepared to 5 = very prepared. M = mean; SD = standard deviation; IQR = interquartile range (Q1–Q3). ^†^ Missing data= 2.

**Table 5:** Approaches to the treatment of sleep difficulties.

	N	%
**In your opinion, how important is it for sleep difficulties to be addressed in a course of psychotherapy?**		
From 1 = not important to 5 = very important		
1	0	0.0
2	4	1.9
3	33	15.5
4	80	37.6
5	96	45.1
**If a patient has sleep difficulties, do you dedicate one or more session to treating them?**		
Never	23	10.8
Sometimes	130	61.0
Always	55	25.8
No clinical activity	5	2.3
**What type of intervention/procedure do you apply more frequently to address sleep difficulties in your patients? ^†^**		
Referral to primary care physician or specialist (e.g., neurologist)	71	33.3
I apply cognitive behavioral treatment for insomnia	60	28.2
I share sleep hygiene rules	155	72.8
I prescribe relaxation techniques in the evening	132	62.0
I share behavioral suggestions (e.g., engage in regular physical activity)	136	63.8
No current clinical activity	7	3.3
Other	13	6.1

^†^ Multiple choices.

**Table 6:** Prevalence of reporting to be familiar or very familiar with different CBT-I techniques in subgroups.

			Experience						Frequency of Dedicating Sessions to Sleep Difficulties ^†^					
	Total		No Experience		0–5 years		More than 5 years		Never		Sometimes		Always	
			(n = 39)		(n = 74)		(n = 100)		(n = 23)		(n = 130)		(n = 55)	
	n	%	n	%	n	%	n	%	n	%	n	%	n	%
Sleep Hygiene	157	73.7	19	48.7	54	73.0	84	84.0	11	47.8	91	70.0	54	98.2
Sleep Restriction	64	30.0	6	15.4	20	27.0	38	38.0	4	17.4	32	24.6	28	50.9
Stimulus Control	97	45.5	10	25.6	25	33.8	62	62.0	5	21.7	53	40.8	39	70.9
Cognitive Control	86	40.4	8	20.5	28	37.8	50	50.0	5	21.7	44	33.8	37	67.3
Paradoxical Intention	37	17.4	2	5.1	14	18.9	21	21.0	1	4.3	18	13.8	18	32.7
Relaxation techniques	153	71.8	20	51.3	53	71.6	80	80.0	12	52.2	91	70.0	50	90.9
Imagination techniques	87	40.8	10	25.6	28	37.8	49	49.0	7	30.4	48	36.9	32	58.2
Mindfulness applied to insomnia	83	39.0	7	17.9	30	40.5	46	46.0	2	8.7	47	36.2	34	61.8
Extinction (infancy)	30	14.1	2	5.1	17	23.0	11	11.0	0	0.0	15	11.5	15	27.3
Stimulus control for children	43	20.2	5	12.8	21	28.4	17	17.0	0	0.0	23	17.7	20	36.4

^†^ Total does not include n = 5 responding that they did not practice at the moment.

## Data Availability

The data presented in this study are available on request from the corresponding author. The data are not publicly available due to ethical restrictions.
